# 90 days impacts of remote ischemic preconditioning on patients undergoing open total aortic arch replacement: a post-hoc analysis of previous trial

**DOI:** 10.1186/s12871-020-01085-9

**Published:** 2020-07-09

**Authors:** Yimeng Chen, Guyan Wang, Hui Zhou, Lijing Yang, Congya Zhang, Xiying Yang, Guiyu Lei

**Affiliations:** 1 Department of Anesthesiology, Fuwai Hospital, Chinese Academy of Medical Sciences and Peking Union Medical College, National Center for Cardiovascular Diseases, State Key Laboratory of Cardiovascular Disease, Belishi road 167, Xicheng District, Beijing, 100037 China; 2 Department of Anesthesiology, Beijng Tongren Hospital, Capital Medical University, No. 1 Dongjiaominxiang, Dongcheng District, Beijing, 100730 China; 3 Department of Anesthesiology, Ruijin Hospital, Shanghai Jiaotong University School of Medicine, Shanghai, China

**Keywords:** Remote ischemic preconditioning, Open total aortic arch replacement, Major adverse kidney events

## Abstract

**Background:**

In the previous randomized controlled trial by our research group, we evaluated the effect of remote ischemic preconditioning (RIPC) in 130 patients (65 per arm) on acute kidney injury (AKI) within 7 days of open total aortic arch replacement. Significantly fewer RIPC-treated patients than sham-treated patients developed postoperative AKI, and, epically, RIPC significantly reduced serious AKI (stage II–III). However, the long-term effect of RIPC in patients undergoing open total aortic arch replacement is unclear.

**Methods:**

This study was a post-hoc analysis. We aimed to assess the roles of RIPC in major adverse kidney events (MAKE), defined as consisting persistent renal dysfunction, renal replacement therapy and mortality, within 90 days after surgery in patients receiving open total aortic arch replacement.

**Results:**

In this 90-day follow-up study, data were available for all study participants. We found that RIPC failed to improve the presence of MAKE within 90 days after surgery (RIPC: 7 of 65[10.8%]) vs sham: 15 of 65[23.1%]; *P* = 0.061). In those patients who developed AKI after surgery, we found that the rate of MAKE within 90 days after surgery differed between the RIPC group and the sham group (RIPC: 4 of 36[11.2%]; sham: 14 of 48[29.2%]; *P* = 0.046).

**Conclusions:**

At 90 days after open total aortic arch replacement, we failed to find a difference between the renoprotective effects of RIPC and sham treatment. The effectiveness or ineffectiveness of RIPC should be further investigated in a large randomized sham-controlled trial.

**Trial registration:**

This study was approved by the Ethics Committee of Fuwai Hospital (No. 2016–835) and our previous study was registered at clinicaltrials.gov before patient enrollment (NCT03141385; principal investigator: G.W.; date of registration: March 5, 2017).

## Background

Acute kidney injury (AKI) is a severe complication after cardiac surgery, causing considerable increases in morbidity, mortality, and health care costs for patients [1]. Open total aortic arch replacement is characterized by a long intraoperative duration and extended hypothermic circulatory arrest time. The incidence of AKI in patients is significantly higher following open total aortic arch replacement than following other cardiac surgical procedures, reaching as high as 77.6% according to our previous retrospective analysis [2]. The therapeutic options for AKI following open total aortic arch replacement remain a significant challenge.

Studies have reported the protective roles of remote ischemic preconditioning (RIPC) on distant organs via alternation between ischemia and reperfusion [3] . However, the efficacy of RIPC is controversial. Several large trials published in high-profile journals (e.g., *NEJM*, *Anesthesiology*) have reported conflicting results.

Meybohm et al. [4] (Remote Ischemic Preconditioning for Heart Surgery trial) and Hausenloy et al. [5] (the Effect of Remote Ischemic Preconditioning on Clinical Outcomes in Patients Undergoing Coronary Artery Bypass Graft Surgery trial) did not find renoprotective effects of RIPC, and the result was maintained up to 1 year after the surgery in the Remote Ischemic Preconditioning for Heart Surgery study [6]. However, Zarbock and colleagues demonstrated that RIPC, compared with sham treatment, significantly reduced the rate of AKI (*P* = 0 .02) and actually reduced the proportions of stage II–III cases (*P* = 0 .02) following cardiac surgery [7]. In subsequent follow-up analyses of their trial, they found that RIPC markedly reduced the 90-day incidence of composite major adverse kidney events (MAKE), defined as consisting of mortality, need for renal replacement therapy, and persistent renal dysfunction [8].

Limited long-term data are currently available on the impacts of RIPC among patients after complex heart surgery, such as the currently available open total aortic arch replacement. However, long-term data beyond AKI are essential to demonstrate a meaningful effect [9]. The U.S. Food and Drug Administration (FDA) states that in addition to reducing inpatient AKI, a treatment must successfully intervene in AKI to improve long-term renal function or hard endpoints such as chronic kidney disease (CKD) or mortality [10]. In a previous randomized controlled trial by our research group, we evaluated the effect of RIPC in 130 patients (65 per arm) on AKI within 7 days of open total aortic arch replacement [11]. We demonstrated that significantly fewer patients developed postoperative AKI with RIPC compared with sham (*P* = 0.028), and epically, RIPC significantly reduced serious AKI in a previous study (*P* = 0.001) [11].

Therefore, in this study, we aimed to assess the roles of RIPC in MAKE 90 days after surgery in patients receiving open total aortic arch replacement.

## Methods

### Trial design

This article is reported as per Consolidated Standards of Reporting Trials (CONSORT) reporting guidelines (www.consort-statement.org) (Supplementary Material Table S1).

This study is a follow-up to our previous study. We conducted an explorative.

post-hoc analysis of our previous single-center, randomized, sham-controlled clinical trial [11]. As already described elsewhere [11], this study was conducted in a high-volume center for thoracic aortic surgery from April 2017 to March 2018. The Ethics Committee of Fuwai Hospital approved the study (No. 2016–835). All participants provided written informed consent before randomization. The clinical trial was registered at clinicaltrials.gov before patient enrollment ((NCT03141385). All procedures were conducted in accordance with the ethical standards of the 1964 Declaration of Helsinki and its later amendments.

In our previous study, one hundred thirty participants who underwent open total aortic arch replacement were randomly assigned in a 1:1 ratio to undergo either RIPC or sham RIPC (control group).Randomization, treatment assignment, and implementation of two interventions by clinical researchers who are not involved in data collection and analysis.

### Procedures and interventions

This procedure was performed to treat extensive aortic dissections or aneurysms and replace the arch using a tetrafurcate graft with stented elephant trunk implantation. All procedures were performed through a standard median sternotomy under cardiopulmonary bypass (CPB). After induced anesthesia, RIPC and sham RIPC were conducted before skin incision. In the RIPC group, four cycles of 5-min inflation at 200 mmHg or at least 50 mmHg higher than systolic pressure in the right upper limb were performed, and 5-min reperfusion with the cuff deflation was subsequently conducted. In the sham group, sham RIPC was performed, comprising four cycles of pseudo-ischemia and reperfusion (5-min blood pressure cuff inflation at 20 mmHg) in the right upper limb and a subsequent 5-min cuff deflation.

### Data collection

Surgeons in our clinical center routinely advise patients return to the hospital for 3 months of follow-up after surgery to observe whether the patients recover well. Eighty percent of patients in our study came to our clinical center for 3 months of postoperative follow, which was conducted with related blood biochemical examination and imaging examination. Thus, the data of 80 % of patients were obtained from hospital records, and the data of the other 20 % of patients were obtained by phone interviews with patients and family members 90 days after surgery. A variation of ±3 days was allowed for logistical reasons.

### Endpoints

AKI is a serious postoperative complication, but CKD is even more severe. In order to observe the renal status of patients after AKI, a common indicator of general acceptance and chronic renal insufficiency must be established. Emerging demand for dialysis following AKI and persistent renal dysfunction (worsened CKD) after AKI herald subsequent possible major morbidity and death. In addition, considering that death after AKI is actually more common than dialysis and is the ultimate major morbid outcome after AKI, its inclusion in a composite endpoint is practically mandatory [12]. Thus, the composite outcome of death, new dialysis, and persistent renal dysfunction constitutes MAKE [10]. MAKE should be assessed at specific time intervals following AKI diagnosis, of which 90 days may be the best endpoint because that is typically the threshold when CKD is diagnosed after AKI [13].

The key endpoint of this study was the composite outcome MAKE consisting of all-cause mortality, persistent renal dysfunction and dialysis in all patients within 90 days postoperatively. The secondary endpoints were all-cause mortality, persistent renal dysfunction and dialysis in all patients within 90 days postoperatively. Other secondary endpoints included the composite outcome MAKE in patients who developed AKI after open total aortic arch replacement within 90 days postoperatively.

Specifically, the definition of clinical endpoints of the Zarbock et al. study [8] have been used for reference for our study, because the study also examined the 90-day impacts of RIPC on kidney function in patients at high AKI risk (Cleveland Clinic score ≥ 6) undergoing cardiac surgery. The definition of persistent renal dysfunction was serum creatinine levels greater than or equal to 0.5 mg/dl (44 μmol/L) over baseline serum creatinine in patients not receiving dialysis or dialysis dependency [8]. Patients who died within 90 days could not be assessed for persistent renal dysfunction or dialysis.

### Statistical analysis of the data

The key endpoint of and the secondary endpoints in this study were examined using a 2-sided Pearson’s χ^2^ test or Fisher’s exact test if proper between patients treated with and without the RIPC technique.

The subgroup analysis of the key endpoint using propofol for anesthesia maintenance was examined using a 2-sided Pearson’s χ^2^ test or Fisher’s exact test if proper between patients treated with and without the RIPC technique.

The sample size of the previous clinical trial of 7-days post operation was based on our retrospective cohort study [2], we estimated that 75% of participants in the sham group would develop into AKI after surgery. The expected absolute risk reduction for AKI was 25% according to the result of the pilot study. Accordingly, to detect a 25% absolute risk reduction in the primary end point in the RIPC group (from 75 to 50%), with a power of 80% and a significance level of 5%, the result of the sample size was total 116 (total 130 including drop-out data).

A *p* value < 0.05 indicated significance. SPSS version 20.0 (IBM Corp, Armonk, NY) was employed for statistical analysis. All results were considered explorative.

## Results

**Fig. 1:**
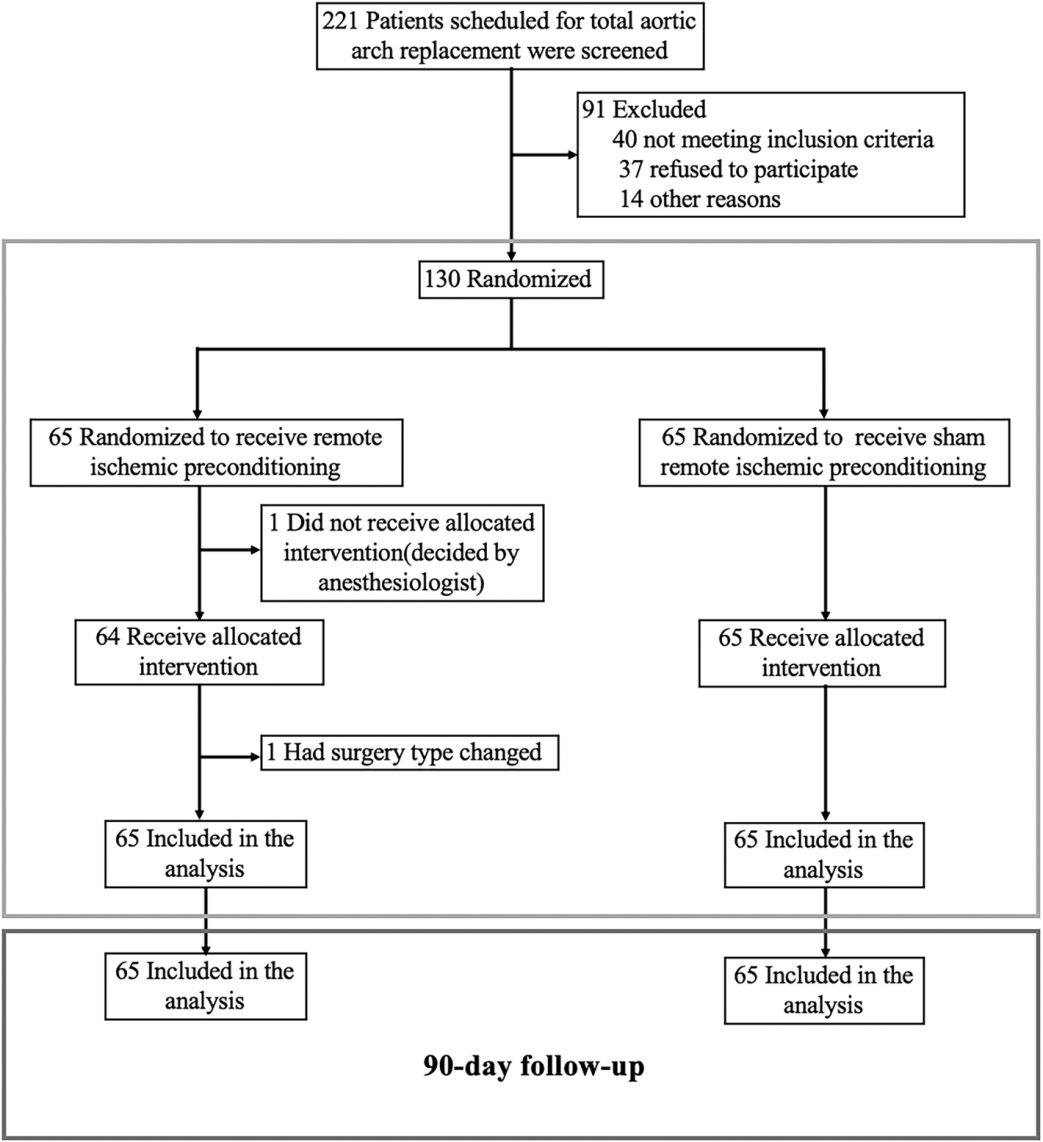
Patient enrollment and allocation to the remote ischemic preconditioning (RIPC) and control (sham) arms. The first part (light blue box) shows the recruitment of our initial trial [11], while the second part (dark blue box) shows the analyzed cohort of this post-hoc analysis

In total, 130 patients were included in our 90-day analysis, and data were available for all study participants, as shown in Fig. 1. We have previously revealed the baseline and operative features of patients in the groups, and there were no relevant imbalances between the two groups at baseline [11].

No significant differences could be found between the RIPC group (7 of 65[10.8%]) and sham group (15 of 65[23.1%]; *P* = 0.061) on MAKE at 90 days postoperatively. In addition, persistent renal dysfunction (RIPC 3 of 63 [4.8%] vs sham 5 of 60 [8.3%]; *P* = 0.662), dialysis dependence (RIPC 2 of 63 [3.2%] vs sham 5 of 60 [8.3%]; *P* = 0.398) and mortality (RIPC 2 of 65 (3.1%) vs sham 5 of 65 [7.7%]; *P* = 0.437) were not significantly different between the two groups (Table 1).

**Table 1:** The key endpoint: major adverse kidney events of 90-day in all patients

	n	RIPC	Sham	*P* value
MAKE, n (%)	65/65	7 (10.8)	15 (23.1)	0.061
Persistent renal dysfunction ^b^, n (%)	63/60	3 (4.8)	5 (8.3)	0.662^a^
Dialysis dependence^b^, n (%)	63/60	2 (3.2)	5 (8.3)	0.398^a^
Mortality, n (%)	65/65	2 (3.1)	5 (7.7)	0.437^a^

*MAKE* major adverse kidney events, *RIPC* remote ischemic preconditioning

^a^Fisher exact test was used due to expected counts less than 5 in the cross-table

^b^Excluding patients who died

In the 84 of 130 (64.6%) patients who developed AKI after open total aortic arch replacement (RIPC 36 [55.4%] vs sham 48 [73.8%]), we found a difference between the RIPC group and the sham group in terms of MAKE at 90 days after surgery (4 of 36[11.1%]) and the sham group (14 of 48[29.2%]; *P* = 0.046), while persistent renal dysfunction (RIPC 2 of 34 [5.9%] vs sham 5 of 41 [12.2%]; *P* = 0.591), dialysis dependence (RIPC 1 of 34 [2.9%] vs sham 4 of 41 [9.8%]; *P* = 0.476) and mortality (RIPC 1 of 36 [2.8%] vs sham 5 of 48 [10.4%]; *P* = 0.358) were similar in both groups (Table 2).

**Table 2:** Composite endpoint major adverse kidney events of 90-day in patients with AKI

	n	RIPC	Sham	*P* value
MAKE, n (%)	36/48	4 (11.1)	14 (29.2)	0.046
Persistent renal dysfunction ^b^, n (%)	34/41	2 (5.9)	5 (12.2)	0.591^a^
Dialysis dependence^b^, n (%)	34/41	1 (2.9)	4 (9.8)	0.476^a^
Mortality, n (%)	36/48	1 (2.8)	5 (10.4)	0.358^a^

*MAKE* major adverse kidney events, *RIPC* remote ischemic preconditioning

^a^Fisher exact test was used due to expected counts less than 5 in the cross-table

^b^Excluding patients who died

This effect of RIPC on the key endpoint was consistent in the subgroup analysis of patients using propofol for maintenance of anesthesia (*P* = 0.774) (Table 3).

**Table 3:** Subgroup analysis of the key endpoint: propofol with or without volatile agents for anesthesia maintenance during surgery

Subgroup	RIPC	Sham	*P* value
	*Number (%) of patients who met MAKE at 90d*		
Anesthetics for maintenance			
Propofol with or without volatile agents	4 (66.7%)	11 (84.6%)	0.774

*MAKE* major adverse kidney events; *RIPC* remote ischemic preconditioning

## Discussion

According to our results, the current study did not find evidence to support an effect of RIPC on MAKE (mortality, need for renal replacement therapy, and persistent renal dysfunction) evaluated at 90 days postoperatively. We found that in patients who developed AKI after surgery, RIPC significantly reduced MAKE (*P* = 0.046) within 90 days postoperatively. In contrast, our previous randomized, sham-controlled study found evidence of a reduction in AKI with RIPC within 7 days postoperatively (*P* = 0.028), epically RIPC significantly reduced serious AKI (stage II–III) (*P* = 0.001).

This inconsistency may be due to several causes. First of all, patients in our study were relatively young (RIPC: 47.8 ± 10.4 years; sham:45.4 ± 10.2 years) with a relatively good preoperative health status. From our clinical experience and some previous studies, patients with preexisting renal injury have worse outcomes compared with patients with normal kidney function in terms of mortality and the need for renal replacement therapy [14–16]. The 90-day follow-up study of Zarbock et al. [8] had the same clinical endpoint as our study, but the results of the two studies were contrary. A possible explanation was that 74 patients had CKD before surgery in the study of Zarbock et al. [7], whereas there was only one patient with CKD before surgery in our study [11]. In addition, patients with comorbidities such as hypertension and diabetes mellitus make it difficult to recover from AKI [17]. The proportion of patients with comorbidities (diabetes mellitus, hypertension, etc.) in our research was much less than that in the Zarbock et al. study. Patients with chronic diseases, such as diabetes mellitus and hypertension, may have diminished glomerular reserve [18]. Diabetes mellitus itself is the main cause of CKD. Therefore, patients in proper preoperative status in our study recovered easily at 90 days after surgery.

Second, RIPC may have a diminished long-term protective effect in patients undergoing open total aortic arch replacement. We enrolled only patients who underwent open total aortic alone or combined with other types of surgery. Patients often have potentially unstable cardiovascular conditions before surgery. Open total aortic arch replacement is a complex type of cardiac surgery with a potentially unstable perioperative course and obvious hemodynamic fluctuations. Studies have suggested that patients suffer from prerenal AKI, which could be caused by decreased renal perfusion due to hypotension and cardiovascular instability [19]. We speculated that the AKI following open total aortic arch replacement was mostly prerenal AKI, and doctors in the intensive care unit usually compensated for the insufficiency of the blood volume at our clinical center. Furthermore, CPB, hypothermia and cardioplegia itself are known to have renoprotective effects during the perioperative period, and RIPC may not have obvious renoprotection effects in the long term [20]. In summary, in terms of patients receiving open total aortic arch replacement, RIPC in our study may not affect 90-day MAKE, possibly because timely treatment and other protective measures (CPB, etc.) are applied during the perioperative period.

Third, we included a relatively small sample of patients. The findings were exploratory. This study was considered very different from short-term studies on renal outcomes. Despite the lack of statistical significance for MAKE (*P* = 0.061), patients in the sham arm died more than twice as those in the RIPC group, and the key endpoint of MAKE was almost twice as common in the sham group as in the RIPC group. Interestingly, in patients who developed AKI after surgery, RIPC significantly reduced MAKE (*P* = 0.046). RIPC may improve renal recovery of patients with AKI at 90 days after surgery. This study will shape our future research efforts to conduct a large randomized sham-controlled study concerning the long-term efficacy of RIPC.

The negative results of this study may have been relatively unaffected by the use of propofol. Propofol, which was reported to attenuate the protective effect of RIPC [21], was used in our study. Nearly 90% of the included patients were treated with propofol alone or combined with sevoflurane for anesthesia maintenance. However, in the subgroup analysis of propofol anesthesia recipients in terms of the key endpoint (MAKE) in our study [11], we found propofol may not affect the results(*P* < 0.774). The mechanism of how propofol affects the renoprotective effects of RIPC is unclear. Two basic studies showed that propofol reversed myocardial protection afforded by RIPC through inhibition of release or transport of humoral factors in rats model [22, 23]. Propofol may play a protective role in the renal function through humoral factors or other signaling pathways. Although propofol is a short-acting anesthetic, whether the mechanism of propofol having an impact on the renoprotective effects of RIPC still should be further investigated.

The strength of our study includes the relatively long period of follow-up assessing the effect of RIPC treatment on MAKE in a population at very high risk for kidney injury. Our study has some limitations. First, the study is a post-hoc analysis, and the sample size of patients was relatively small. Thus, the study faces a risk of type I and II errors. The findings were exploratory and should be interpreted with caution. Second, the diagnosis of AKI was principally based on an increase in serum creatinine, which may not accurately reflect real changes in the glomerular filtration rate. At present, however, serum creatinine remains the most widely used biomarker to evaluate kidney function [24]. Third, our study did not include any mechanistic exploration of the effects of RIPC application in patients undergoing open total aortic arch replacement.

## Conclusions

The present study is the first to demonstrate the effects of RIPC in patients during open total aortic arch replacement on 90-day clinical outcomes. We failed to find a difference in 90-day postoperative renoprotective effects between the RIPC and sham-treated groups among patients receiving open total aortic arch replacement. The effectiveness or ineffectiveness of RIPC should be further investigated in a large randomized sham-controlled trial.

## Supplementary information

**Additional file 1.** The CONSORT Statement checklist.

## Data Availability

The datasets used or analyzed during the current study are available from the corresponding author on reasonable request.

## Abbreviations

RIPC: Remote ischemic preconditioning
AKI: Acute kidney injury
MAKE: Major adverse kidney events
CKD: Chronic kidney disease
CPB: Cardiopulmonary bypass
